# Optical Camera Communications for IoT–Rolling-Shutter Based MIMO Scheme with Grouped LED Array Transmitter

**DOI:** 10.3390/s20123361

**Published:** 2020-06-13

**Authors:** Shivani Rajendra Teli, Vicente Matus, Stanislav Zvanovec, Rafael Perez-Jimenez, Stanislav Vitek, Zabih Ghassemlooy

**Affiliations:** 1 Faculty of Electrical Engineering, Czech Technical University in Prague, Technicka, 16627 Prague, Czech Republic; xzvanove@fel.cvut.cz (S.Z.); viteks@fel.cvut.cz (S.V.); 2 Institute for Technological Development and Innovation in Communications, Universidad de Las Palmas de Gran Canaria, 35001 Las Palmas, Spain; vicente.matus@ulpgc.es (V.M.); rperez@idetic.eu (R.P.-J.); 3 Optical Communications Research Group, Northumbria University, Newcastle-upon-Tyne NE1 7RU, UK; z.ghassemlooy@northumbria.ac.uk

**Keywords:** optical camera communications, rolling-shutter camera, Internet of things, multiple-input multiple-output, light-emitting diodes

## Abstract

In optical camera communications (OCC), the provision of both flicker-free illumination and high data rates are challenging issues, which can be addressed by utilizing the rolling-shutter (RS) property of the image sensors as the receiver (Rx). In this paper, we propose an RS-based multiple-input multiple-output OCC scheme for the Internet of things (IoT) application. A simplified design of multi-channel transmitter (Tx) using a 7.2 × 7.2 cm^2^ small 8 × 8 distributed light emitting diode (LED) array, based on grouping of LEDs, is proposed for flicker-free transmission. We carry out an experimental investigation of the indoor OCC system by employing a Raspberry Pi camera as the Rx, with RS capturing mode. Despite the small area of the display, flicker-free communication links within the range of 20–100 cm are established with data throughput of 960 to 120 bps sufficient for IoT. A method to extend link spans up to 1.8 m and the data throughput to 13.44 kbps using different configurations of multi-channel Tx is provided. The peak signal-to-noise ratio of ~14 and 16 dB and the rate of successfully received bits of 99.4 and 81% are measured for the shutter speeds of 200 and 800 µs for a link span of 1 m, respectively.

## 1. Introduction

The fifth-generation (5G) telecommunication standards have set the new platform for mobile wireless networks, rather than just extending the transmission capacity and reliability of the 4G network [1]. With the three main focuses on capacity enhancement, massive connectivity, and ultra-high reliability (i.e., low latency), 5G development stems largely from the increasing number of users and smart devices within the context of Internet of things (IoT)-based smart environments being connected to the cellular networks [2]. In future smart environments, such as homes, offices, cities, etc., there will be a growing need for the communications networks that can facilitate connectivities between a large number of devices or sensors and the end-users [3]. This will mean access to substantial transmission resources (i.e., bandwidth), and thus a paradigm shift in the way the wireless transmission resources are utilized effectively.

To address this paradigm, a number of technologies have been proposed, including millimeter-wave [4], massive multiple-input multiple-output (MIMO) [5], small cell [6], and optical wireless communications (OWC) [7,8], in order to meet the requirements of 5G-IoT. The popularity of the latter is due mainly to its inherent advantages of safety, security, low cost, and large transmission bandwidth, which are essential in IoT applications, particularly in indoor environments [8]. In indoor environments, the OWC technologies of visible light communications (VLC) and optical camera communications (OCC) have been widely considered and investigated [8,9]. While VLC can support major features of 5G in terms of the data rates, OCC can offer a promising solution. The VLC technology, which uses light-emitting diode (LED)-based lights as the transmitter (Tx), has a lower initial deployment cost. The cameras in smartphones (six billion front and rear cameras), and pre-existing camera-based infrastructures, such as traffic lights, security, surveillance, and vehicles can be adopted as the OCC receiver (Rx) effectively [10]. Reflecting on the OCC potential and its availability, the IEEE 802.15.7r1 task group has been established to develop a standard for OCC within OWCs [9]. Various OCC issues and considerations, such as image sensor architecture, synchronization, data rate, perspective distortion, flickering and dimming, MIMO, and diversity are studied in [11]. The use of high-rate Tx’s, such as LEDs, and low-rate Tx’s, such as liquid crystal displays and digital signage, in OCC schemes is also provided in [11]. Although OCC does not support high capacity link connectivities, due to the speed limit of cameras, it can be employed in numerous low data rate applications, such as indoor positioning, mobile robot navigation, vehicular communications, small identification information, and advertisements [8,9,10,11]. Despite lower data rates, OCC is simple to implement, with low path loss, additional imaging functionality, and spatial modulation capability, as compared with high rate VLC links [12].

However, in OCC, the data rate is limited by the frame-rate of the image sensors (ISs). The data rate can be increased by using higher frame rate cameras, which are very costly, and increasing the camera capture speed, which is defined as the physical parameter of the sensor (electronics) and the graphics processor speed in the hardware domain. Therefore, OCC is further extended to offer massive MIMO capabilities in order to increase the data rate, using LED and photodetector (PD) arrays in the form of multiple pixels in ISs for IoT applications, in both indoor and outdoor environments [13]. In addition, hybrid modulations schemes based on the intensity, color, spatial, phase, and frequency are also suggested in [12] as a solution to improve the transmission data rates in OCC.

The IEEE 802.15.7-2018 standard [14] defines new clauses for the physical layer (PHY) types V and VI for OCC links. These PHY layers are mainly intended for use in systems with diffused light sources and video displays with kbps data rates, as well as using complex modulation schemes of spatial two-phase shift keying (S2-PSK), dimmable spatial eight-PSK, undersampled frequency shift on-off keying (UPSOOK), variable transparent amplitude-shape-color, etc. However, the standard clauses on the PHYs V and VI are mainly on the demodulation schemes, and their real applications are still being revised.

In [13], a data rate of 126.72 kbps was achieved, using 192 data-carrying LEDs modulated using color intensity modulation (CIM) and a 330 frames per second (fps) global-shutter (GS) camera-based Rx, which is expensive and not commonly used, over a link span of 1.4 m. However, the Tx was set to the refresh rate (i.e., transmission frequency) of 82.5 Hz, which is still lower than the maximum allowed flickering time period of 5 ms (200 Hz) [14]. A red, green, and blue (RGB) LED-based rolling-shutter (RS) OCC (RS-OCC) utilizing a combination of UPSOOK, wavelength-division multiplexing, and MIMO offering improved space efficiency of 3 bits/Hz/LED was reported in [15]. In [16], a multilevel intensity modulation (IM) RS-based camera detection link with a data throughput of 10 kbps over a transmission range of 2 m was reported in [15,16]. Whereas, a beacon jointed packet reconstruction scheme for mobile-phone-based VLC with commercial white phosphor LEDs and a 60 fps RS camera achieved the net data rate of 10.3 kbps (172 bits/frame observed over a large LED surface) over a transmission distance of 20 cm [17]. In [18], COTS LEDs with raptor code (with linear time encoding and decoding, thus reduced computational complexity and decoding overhead) have been investigated in RS-OCC. Non-line-of-sight MIMO links using Luxeon LEDs based on diffused reflections and space and time division multiple access, as well as the equal gain combining technique, was reported in [19], which achieved flicker-free transmission up to a 10 m link span. However, as listed in the survey in [20], for indoor OCC based IoT applications, low data rates ranging from 15 to 896 bps for transmitting short messages in device-to-device communications over transmission spans of 25 cm to 1 m is more than sufficient. 

Over the past few years, multiple neopixel boards have been used in electronic devices such as screen displays in home automation, advertising, televisions, human interfaces, etc. [21]. These devices can be used as part of the Tx to provide IoT based MIMO-OCC links in smart environments. High-throughput links such as visual MIMO systems for screen-camera communications link were proposed in [22], where the impact of non-linear channel equalization, non-binary channel coding, probabilistic shaping, and non-linear precoding for high-order modulation schemes and respective applications such as inter/intra vehicle communications, near field communication, and augmented reality were investigated. In [22], it was shown that the reliability and throughput of the optical communication links can be improved using various channel coding techniques based on nonbinary low-density parity-check codes [23], polar and turbo codes [24], and advanced modulation schemes, such as chaos and LoRa [25]. As previously mentioned, short-range and low data rates IoT links in indoor environments such as device-to-device communications with short messages, indoor positioning, navigation, small identification information, and communications through advertisements need to be considered [26]. However, the major challenge in implementing such systems is the requirement for flicker-free transmission at lower data rates (i.e., most cases in IoT applications). Therefore, in this paper, we propose a low data rate RS-based MIMO-OCC scheme with grouped LED Tx for indoor IoT environments, which is flicker-free. For this reason, we present a simplified design of MIMO-OCC grouped LED array-based Tx, which uses 64-neopixel LEDs distributed in an 8 × 8 array and a commercial, low-cost Raspberry Pi camera (RaspiCam) as the Rx. The Tx unit is divided into eight different groups, with eight LEDs per group in order to increase the data rate and achieve flicker-free transmission. At the Rx, RaspiCam, with a resolution of 1920 × 1080 pixels and a capture speed of 30 fps, captures the LED array in RS-mode at different shutter speeds (SS) and transmission links *L*. The novelty of this work is on the design of a simplified Tx for multiple channels transmission-based LED grouping with perfect synchronization, and the use of an RS camera for flicker-free transmission in short-range and low data rates IoT applications. The paper gives a detailed analysis on the quality matrix of the captured image in terms of the peak signal-to-noise ratio, and the success rate of received bit sequences with respect to the transmission span and the camera’s SS.

The remainder of the paper is organized as follows: Section 2 describes the proposed RS-based MIMO-OCC scheme using the grouped LED array, while Section 3 shows the experiment setup, followed by the discussion of results. Conclusions are drawn in Section 4.

## 2. Proposed RS-Based MIMO-OCC Scheme with Grouped LED Tx

### 2.1. MIMO-OCC Tx Characterization

**Figure 1:**
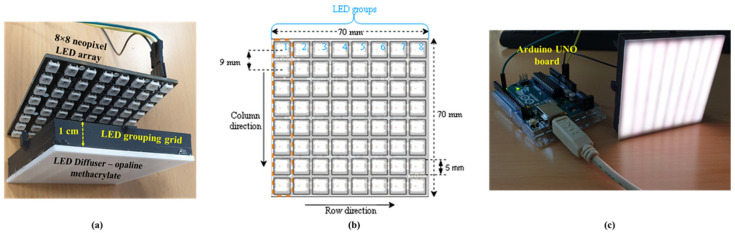
The transmitter (Tx) unit: (**a**) assembled unit with the grid and a diffuser, (**b**) an LED array configuration and (**c**) an Arduino Uno controller board and the LED panel.

A new, simple design of MIMO Tx unit is proposed in this paper, as illustrated in Figure 1. It is composed of a 64-neopixel array with an 8 × 8 small chip-LED, and a 1 cm-thick LED grouping grid, (see Figure 1a,b), which is attached over the Tx LED array. This LED grid is designed to divide a 64-neopixel chip-LED into eight different column-wise groups that are individual transmission channels with eight chip-LEDs per group to allow eight different data transmissions, using a single neopixel LED array. In addition, the LED grid (Figure 1c) is very effective in combating interference due to adjacent LEDs within the groups. The effect of interference with supporting analysis without using the grouping grid to capture the LED array with a GS-based camera Rx was initially reported by the authors in [27]. A 2 mm-thick opaline methacrylate LED diffuser, commonly used, is placed over the Tx [28]. The size of the LED array is 7.2 × 7.2 cm^2^. Since the size of each chip-LED (i.e., 5 × 5 mm^2^) is much smaller than the distance between the adjacent chip-LED (i.e., 9 mm), light from each LED is captured as a discrete image using an IS.

Since both optical and electrical characterization of neopixel RGB LEDs are yet to be investigated, we first provide a characterization of the proposed MIMO Tx in terms of its optical radiation pattern and output optical power–current–voltage (*L*_lux_*-I-V*) curves. The optical radiation pattern of the neopixel LEDs was measured to obtain its spatial intensity distribution for use in analyzing the coverage and signal distribution in VLC and OCC links [8]. The light intensity of LEDs defined in terms of the angle of irradiance *θ* is given by [8]:

(1) $$I(\theta )=\frac{m+1}{2\pi }I(0)\cos^{m}(\theta ),\;\theta =[-\frac{\pi }{2},\frac{\pi }{2}],$$

where *I*(0) is the center luminous intensity of an LED and *m* is Lambertian order given as [8]:

(2) $$m=-\frac{\ln(2)}{\ln[\cos(\theta_{1/2})]}.$$

**Figure 2:**
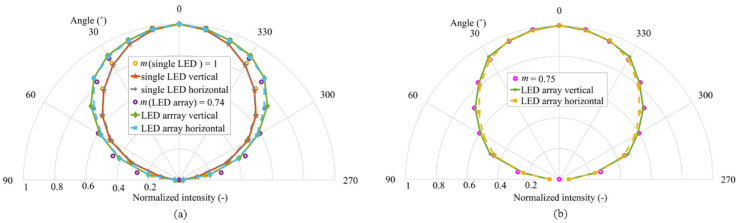
Measured Tx radiation patterns of: (**a**) a single LED in an array and for full LED array; and (**b**) a full LED array with the grid and the diffuser.

A lux meter was used to measure the angular dependence of the luminance of the LED (i.e., single and 8 × 8 horizontal and vertical array). As expected, the profiles for (i) a single LED represent a complete hemisphere close to Lambertian emitter with *m* of 1 (see Figure 2a); (ii) the LED array with no diffuser is broader with *m* of 0.74, due to the 9 mm spacing between the adjacent LED chips; and (iii) the LED array with the diffuser has *m* of 0.75 (see Figure 2b). Note that the measured radiation patterns can be further used to study the proposed MIMO-OCC links with mobility, and in a multi-user scenario. For example, rotation compensation schemes based on different Tx configurations [29] and wide receiver orientations [30] can be employed to ensure the operability of the proposed MIMO-OCC system in IoT environments.

The illuminance levels of the LEDs were measured using a Testo 545 lux meter. Each neopixel in the array draws up to 60 mA of current *I* to turn ON at the maximum brightness. In realistic environments, when the LED panel is to be used as a lamp, luminance can be efficiently controlled by varying switching power supply output current at the LED drive circuit. Therefore, the implementation of dimming techniques can be included as a part of the further extension of the proposed scheme [31,32]. However, increasing the power would induce crosstalk due to illumination from adjacent LEDs in the MIMO Tx [33].

**Figure 3:**
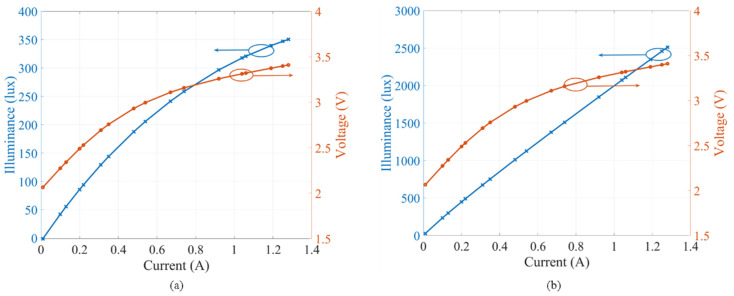
The *L*_lux_-*I*-*V* curves of the multiple-input multiple-output-optical camera communications (MIMO-OCC) Tx unit: (**a**) with the diffuser attached; and (**b**) without the diffuser.

In practical use, it is rare for all pixels to be turned ON at its maximum drawing current, due to the risk of overheating and damaging the LED panel. Therefore, it is recommended to drive each LED with *I*_LED_ = 0.33*I* = 20 mA [21]. Thus, the drive current for the LED array is estimated using the rule of thumb, as given by [21]:

(3) $$I_{\mathrm{LED}\text{-}\mathrm{array}}=\frac{N_{\mathrm{pixels}}\times I_{\mathrm{LED}}}{1000},$$

where *N*_pixels_ = 64 is the total number of neopixel LEDs. In this work, *I*_LED-array_ is set to 1.28 A for measuring the *L*_lux_-*I*-*V* curves, which are depicted in Figure 3a,b for the MIMO-OCC Tx unit with, and without, the diffuser, respectively. Note: (i) the illumination levels are largely reduced, due to the use of the LED grid and the diffuser; (ii) linear *L*_lux_-*I* plots, which are highly desirable in IM VLC systems; and (iii) the neopixels used either as a single LED chip or in an array depict similar optical characteristics to those of commonly used RGB LEDs.

### 2.2. Rolling-Shutter Based MIMO Rx in OCC

The CMOS IS, along with the imaging lens, is composed of a large number of pixels, with each pixel acting as an independent PD. Unlike a conventional PD-based Rx, which cannot be used to separate the mixed signals, CMOS-based IS can capture lights coming from different directions, and project them onto different sections of the IS [13]. Therefore, spatial separation of incoming light signals and their intensities can be measured by obtaining the pixel value for each light source image in the received frame. Image processing can be applied to the received image frames to extract the data from pixelated images [13]. Therefore, CMOS IS can be used as the MIMO-OCC Rx without the need for extended hardware. In [34], the analysis of the MIMO-OCC Rx using Bayer-pattern filters, which can differentiate the incoming signals being transmitted from the MIMO Tx, was reported.

**Figure 4:**
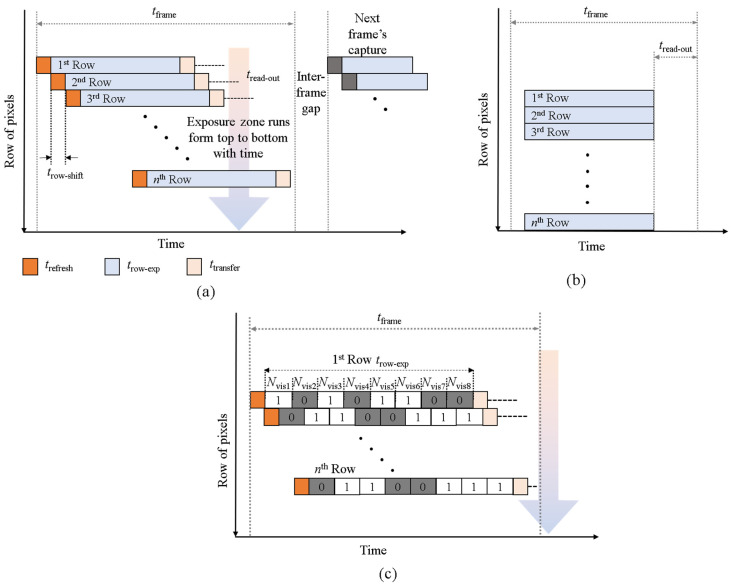
The camera capturing modes in OCC: (**a**) traditional rolling-shutter (RS) mechanism, (**b**) global-shutter (GS); and (**c**) RS-based capturing of the proposed multi-channel Tx.

In an OCC-VLC system, the RS effect of a CMOS IS can be used to achieve flicker-free transmission and increased data rate [16,17,18,19]. In this mode, the camera sequentially integrates light on all pixels at the exposure time *t*_row-exp_, similar to the scanning function, as illustrated in Figure 4a. In RS, the sensor scans the entire image row-by-row (line-wise) and generates a sequential readout. This scan process is governed by the system clock and is limited by the sampling rate of the analog to digital converter module. In GS-based IS, all pixels are exposed to the light simultaneously, i.e., ON or OFF states of the LED in a single frame [35], as illustrated in Figure 4b; while in RS, each row of pixels is exposed to light at a given exposure time *t*_row-exp_, sequentially similar to the scanning function. In RS cameras, the readout time *t*_read-out_ ensures that there is no overlapping of the rows of pixels, and allows multiple exposures in a single captured image. The latter enables multiple LED states to be achieved at the same time in a single frame, as each row is exposed once to the light. Therefore, the captured image of the switched LED is composed of a set of black and white stripes. The proposed MIMO-OCC scheme differs from the RS-based OCC links (see Figure 4c), as in [16,17,18,19]. In traditional RS-based OCC, only a single bit is captured within one exposure time *t*_row-exp_ (see Figure 4a), in contrast to the proposed work, where 8-bit (1-bit per channel) are captured in a single row with time *t*_row-exp_, as depicted in Figure 4c. In addition, the proposed scheme can be used for flicker-free and high data rates transmission by allocating multiple bits per *t*_row-exp_. The widths and the number of strips depend on the data rate (i.e., modulation frequencies) and the camera-LED distance, respectively [16,18].

Note that, in the RS capturing mode, each row starts with a certain delay, which results in the row shift, *t*_row-shift_. The frame time is given as:

(4) $$t_{\mathrm{frame}}\leq N_{\mathrm{row}}\times t_{\mathrm{row}\text{-}\mathrm{shift}}+t_{\mathrm{row}\text{-}\exp},$$

where *N*_row_ is the pixel rows, which are based on the camera resolution. Note, *t*_row-exp_ is the exposure time of the last row per frame (very small value).

### 2.3. System Overview of MIMO-OCC Using Proposed Multi-Channel Tx Design

**Figure 5:**
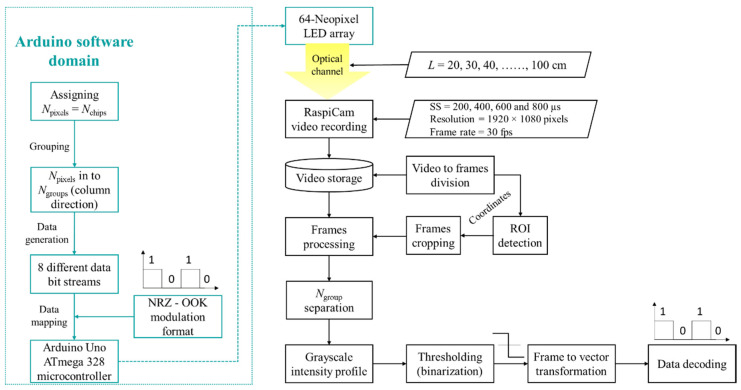
MIMO-OCC data processing flow diagram.

Figure 5 illustrates the flow diagram for the proposed MIMO-OCC, using the 64-neopixel Tx unit and a RS-based RaspiCam as the Rx. The proposed scheme is an initial study to investigate the upper bounds of the system using the proposed Tx unit, therefore, we assume perfect synchronization and line-of-sight transmission. Neopixels are controlled in the Arduino software domain. First, *N*_pixels_ are assigned to *N*_chips_ of neopixels, which are then grouped column-wise into eight *N*_groups_ to form eight different transmission channels within one Tx unit. For data transmission, we have adopted a non-return-to-zero (NRZ) on-off keying (OOK) data format for IM of tri-color (band *i*, *j*, *k*) RGB channels (*P_i_*, *P_j_* and *P_k_*) as *P_i_ + P_j_ + P_k_* = 1 and *P_i_ + P_j_ + P_k_* = 0, which is most commonly used in OCC. The data is generated in the Arduino unit and mapped to the LED addresses with the frequency, *f_s_*, given as:

(5) $$f_{s}={\left(t_{\mathrm{chip}}\right)}^{-1},$$

where *t*_chip_ is the 1-bit time per neopixel chip, and its minimum value is 2.5 ms, due to Arduino hardware limitation to ensure flicker-free transmission at *f_s_* of 400 Hz. The maximum number of visible bits per group in a single frame is given as:

(6) $$N_{\mathrm{visible}}=\left\lfloor \frac{t_{\mathrm{frame}}}{t_{\mathrm{chip}}}\right\rfloor .$$

Note that *N*_visible_ will change with respect to the distance between the source and the camera, as well as camera resolution. Based on *N*_visible_, the data transmission rate is given as:

(7) $$R_{d}=N_{\mathrm{groups}}(\frac{1}{t_{\mathrm{chip}}}),$$

where *N*_groups_ is the number of data transmission channels in the Tx unit (see Figure 1b). 

Table 1 shows the resolutions, frame rate and *N*_visible_ of RaspiCam. For further analysis, we selected a 1920 × 1080 pixel resolution, which is the most commonly used in cameras. 

**Figure 6:**
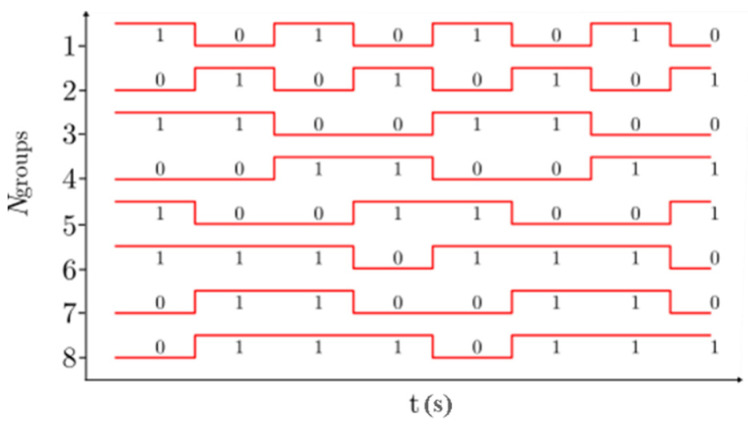
Data bit streams for intensity modulation (IM) of LED groups.

To determine the upper bound of the system, we have selected NRZ-OOK data bit streams for IM of LED groups for transmission over the free space channel. Note that the same data is transmitted by all 8-LEDs per group, as shown in Figure 6.

At the Rx, a RaspiCam for a given SS, resolution, and frame rate is used for capturing the images (i.e., recording a video stream for 3 s) of the IM light sources over an *L* ranging from 20 to 100 cm for post-processing. Note that a smaller image containing the emitter’s signal information is transmitted, in order to speed up the processing time at the Rx. Therefore, the first step represents the detection of the region of interest (ROI) [36]. The obtained coordinates, which define boundaries of the ROI, are used for image cropping. Then, image processing is performed on the cropped images, which are then converted to the grayscale in order to retrieve the intensity profile. The threshold level is set based on the average of the received image intensity profile within the ROI. Following thresholding, binarization of the data frames is performed to convert the frame into vector transformation. This process is performed and applied to the remaining frames for decoding the transmitted data bit streams.

## 3. Experiment Results and Analysis

**Figure 7:**
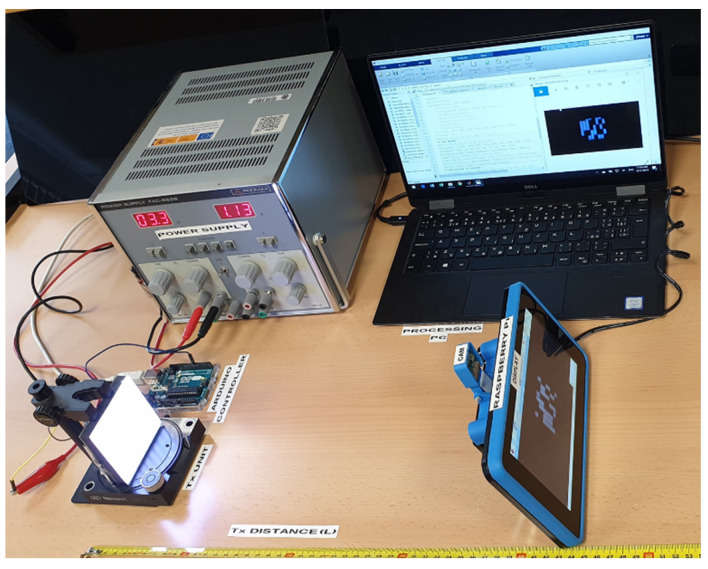
Experiment setup of the MIMO-OCC scheme.

The experimental setup for investigating the proposed MIMO-OCC scheme is shown in Figure 7. The Tx unit is controlled using an Arduino Uno board, which is an open-source microcontroller board based on the ATmega328 [37]. The 64-bit long data stream (i.e., 8-bit per group, see Figure 6) is generated in the Arduino software domain and mapped to each LED address using the Arduino Uno board. 

The key experimental parameters are listed in Table 2. 

The camera used for capturing is the Raspberry Pi official camera (PiCamera V2), which is based on the Sony IMX219 sensor [38]. The RaspiCam is attached to the Raspberry touchscreen display to provide the easy interface and control over the camera capturing modes and settings. For the demonstration of the proposed study, experiments were performed for nine transmission distances and four different values of SS (see Table 2). The NRZ-OOK modulated signal was recorded in the form of a 3 s video stream (90 frames in total).

**Figure 8:**
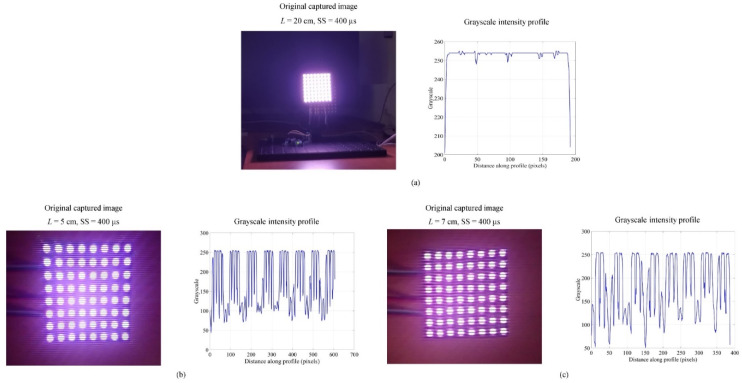
MIMO-OCC performance analysis without grouping grid and diffuser: captured images for data detection and their grayscale intensity profiles for: (**a**) GS captured image for *L* = 20 cm; (**b**) RS captured images for *L* = 5 cm; and (**c**) RS captured images for *L* = 7.

The current proof-of-concept experiments were performed under the ambient light, where we measured the light intensity using a Testo 545 lux meter. The measured light intensity of the Tx with a diffuser was 300 lux at a distance of 50 cm. We also measured reflected lights from walls to be approximately 3.5 lux (±0.5 lux), which is very small compared with the Tx’s illuminance; therefore, the ambient light influence on the integrity of data transmission is insignificant. The experiments were first conducted without a grid and a diffuser. To validate the scheme, a binary bit sequence of 1 and 0 was transmitted via all LEDs in the array. Figure 8 shows the original captured image frames with GS and RS modes, along with respective single row grayscale intensity profiles. Figure 8a shows the captured LED array using GS mode, along with its intensity profile at a maximum *L* of 20 cm. It can be seen that interference from the adjacent LEDs results in the blooming effect and, therefore, causes inter-cluster-interference. It can be seen that LED array captured using RS mode (see Figure 8b) at a minimum *L* of 5 cm, *N*_visible_ is 4 at the surface of each LED. Moving farther away from the Tx, *N*_visible_ is reduced to 3 at *L* of 7 cm (see Figure 8c). This is due to the reduced LED’s surface area of 5 × 5 mm^2^. Moreover, the RS rows also become saturated, which leads to inter-cluster-interference. Therefore, setting threshold levels become problematic, and hence lead to increased bit error rates (see Figure 8b,c). Therefore, further analysis was performed using the proposed LED array configuration shown in Figure 1.

**Figure 9:**
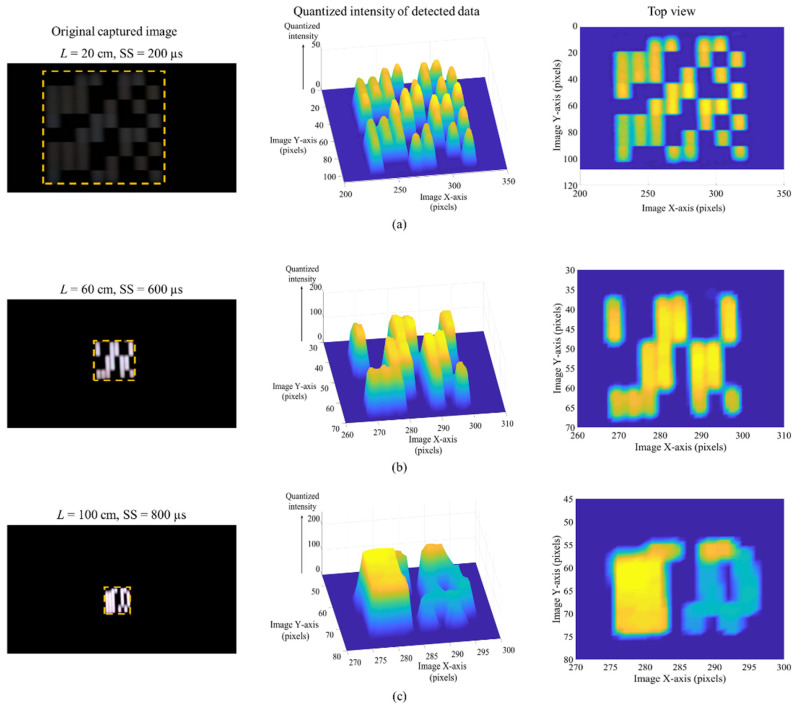
MIMO-OCC performance analysis with grouping grid and diffuser: the quantized intensity profiles of originally captured images for data detection at: (**a**) *L* = 20 cm and SS = 200 µs; (**b**) *L* = 60 cm and SS = 600 µs; and (**c**) *L* = 100 cm and SS = 800 µs.

Figure 9 shows the original captured image frames with grouping grid and diffuser, quantized intensity of the detected data, and the top view of intensity profiles for *L* of 20, 60, and 100 cm, and SS of 200, 600, and 800 µs. The received intensity distribution within the image frame is shown in the form of quantized intensity profiles of the captured original images. These intensity profiles play an important role in determining the higher and lower intensities representing 1 and 0 bits in the received image frames for further thresholding and demodulation [39]. The dotted yellow box in the original image frames is the ROI, which fills only the captured Tx within the full image frame. The clear and sharp distinction between data lines of the adjacent *N*_groups_ can be seen at SS of 200 µs (see Figure 9a), while the lines get saturated for higher values of SS (see Figure 9b,c), which affects data demodulation (i.e., a higher number of bit error). Note that the camera’s SS can be used to combat the effect of ambient lights, where lower and higher SS results in lower and higher levels of light to pass through the camera lens, as seen in the captured images in Figure 9. 

Based on the received bits in the image frames, the data throughput is given as:

(8) $$\mathrm{Data}\;\mathrm{throughput}=N_{\mathrm{groups}}\times N_{\mathrm{visible}}(\frac{\mathrm{fps}}{2}),$$

**Figure 10:**
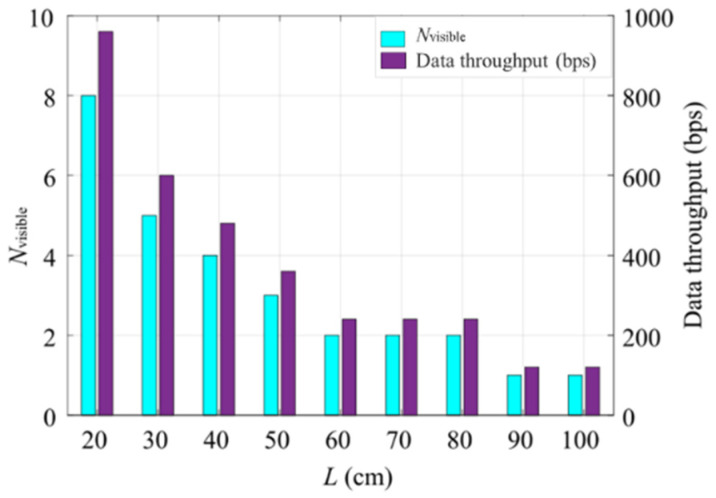
MIMO-OCC performance analysis: the data throughput with respect to *N*_visible_ bits.

Figure 10 illustrates the data throughput and *N*_visible_ with respect to *L*. Note that *N*_visible_ is the number of visible bits in each group. The maximum data throughput of 960 bps is observed at the minimum distance of 20 cm, where 8 full bits are visible in each group. The Tx illumination surface reduces with the increased *L*, thus resulting in reduced *N*_visible_ and the data throughput. For *L* of 100 cm, only one full bit is visible, therefore the data throughput is reduced to 120 bps. 

Table 3 shows the predicted data throughput based on the approximation of different Tx configurations, *L*, *N*_groups_, and *N*_visible_. It can be seen that increasing the number of LEDs leads to a larger surface area of the Tx, thus limiting higher values of *N*_visible_. For example, for a Tx using a 24 × 24 LED array, 24 *N*_groups_ can be formed, which will increase the data throughput by up to 7.92 and 0.360 kbps for *L* of 60 and 160 cm, respectively; while for a Tx with a 32 × 32 LED array, 32 *N*_groups_ can be formed, which will increase the data throughput by up to 13.44 and 0.480 kbps for *L* of 80 and 180 cm, respectively.

Since in OCC the data is captured in the form of a two-dimensional image, a conventional signal-to-noise ratio (SNR) measurement cannot fully reflect the quality of the link. Therefore, we have adopted peak signal-to-noise ratio (PSNR), which is widely used as a quality metric in image processing systems. To compute the PSNR, the mean squared error between the transmitted and received images is given by [39,40]:

(9) $$\mathrm{MSE}=\frac{\sum_{m=1}^{H}\sum_{n=1}^{W}{\left[I_{Tx}(m,n)-I_{Rx}(m,n)\right]}^{2}}{N_{\mathrm{column}}\times N_{\mathrm{row}}},$$

where *I*_Tx_(*m*, *n*) and *I*_Rx_(*m*, *n*) are the intensity levels within the ROI of transmitted and received images of size height (*H*) × width (*W*), and *N*_column_ and *N*_row_ are the number of columns and rows of the images, respectively. The PSNR is then given as:

(10) $$\mathrm{PSNR}=10\log_{10}(\frac{R^{2}}{\mathrm{MSE}}),$$

where *R* is the maximum span of input data (e.g., in the current scheme, the input image has an 8-bit unsigned integer data type; therefore, *R* = 255).

**Figure 11:**
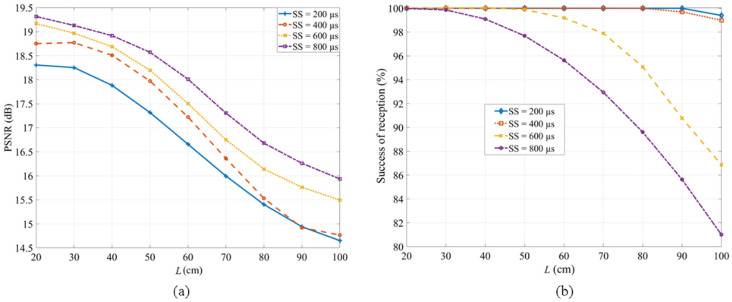
MIMO-OCC performance analysis: (**a**) the peak signal-to-noise ratio (PSNR); and (**b**) the percentage success of received bits with respect to *L*.

Figure 11a shows the performance of the OCC link in terms of PSNR with respect to *L* for different values of SS. As shown, PSNR increases with SS and decreases with the link span. This is due to the fact that the images of captured Tx at higher SS are more saturated, compared with those captured at lower SS (see Figure 9). The PSNR values of ~14 and ~16 dB are measured for SS values of 200 and 800 µs, respectively, for *L* of 100 cm, increasing by 4 and 3 dB for *L* of 20 cm for the same SS values, respectively.

## 4. Conclusions

This paper demonstrated the experimental implementation of an RS acquisition-based camera capturing in MIMO-OCC, employing an LED array in an indoor static environment. The neopixel’s light source could be used either as a single LED chip or an array display with similar optical characteristics to those of commonly used RGB LEDs. We showed the transmission spans of 7 cm and 1 m using the Tx without, and with, the LED grid and a diffuser, respectively. The proposed system with the multi-channel LED array-based Tx offered the same performance at the SS of 200 and 400 µs over an *L* of 80 cm. However, at higher SS, the captured images were saturated, which resulted in increased PSNR and reduced percentage success of received bits. The maximum PSNR values of ~18 and ~19 dB and a 100% success rate of received bits were measured for the SS of 200 and 800 µs at the link span of 20 cm. A theoretical approximation (see Table 3) to further extend the *L* up to 1.8 m and the data throughput up to 13.44 kbps within the proposed MIMO-OCC using a grouped LED array by employing a Tx with a larger illuminating surface (large size) for practical indoor environments was provided. We conclude that the proposed Tx design can provide flicker-free transmission by employing multiple channels Tx with perfect inter-LED synchronization and an RS-based camera for use in short-range and low data rates indoor IoT applications, such as display-to-camera communications. The complexity of the proposed RS-based MIMO-OCC scheme will be further investigated for its implementation in practical scenarios, such as mobility, rotational support, and multiuser, based on ROI detection and bit or image pattern recognition within neural network algorithms.

## Figures and Tables

**Table 1:** RaspiCam resolutions.

Resolution (pixels)	Frame Rate (fps)	*N*_visible_ (bits)
1920 × 1080	30	8
3280 × 2464	15	22
1640 × 1232	40	10
1640 × 922	40	8
1280 × 720	90	6
640 × 480	200	4

**Table 2:** Key parameters of the experiment setup.

Parameter	Value
RaspiCam chip size	5.09 mm (H) × 4.930 mm (W) Diagonal: 4.60 mm
RaspiCam resolution	1920 × 1080 pixels
Raspberry display size	7 ” (diagonally)
Raspberry display resolution	800 × 400 pixels
*t*_chip_	2.5 ms
*f_s_*	400 Hz
RaspiCam frame rate	30 fps
*N*_row_	1080 pixels
*N*_groups_	8 LED groups with 8LED/group
*t*_frame_	0.216 ms
SS	200, 400, 600 and 800 µs
*R_d_*	3.2 kbps
*L*	20–100 cm

**Table 3:** Theoretical (approximation) data throughput based on different Tx configurations.

Number of Neopixels	*N*_groups_	*L* (cm)	*N*_visible_	Data Throughput (kbps)
16 × 16	16	40–140	14 (max)–1 (min)	3.36 (max)–0.240 (min)
24 × 24	24	60–160	22–1	7.92–0.360
32 × 32	32	80–180	28–1	13.44–0.480
